# N‐Glycosylation Modification of Fzd4 Is Essential for the Fzd4‐Wnt‐β‐Catenin Signalling Axis

**DOI:** 10.1111/jcmm.70539

**Published:** 2025-04-14

**Authors:** Tianyi Ji, Xiangying Li, Jiachen Li, Guan Wang

**Affiliations:** ^1^ College of Acupuncture and Tuina Liaoning University of Traditional Chinese Medicine Shenyang China; ^2^ Endocrinology Department Shenyang Fifth People's Hospital Shenyang China; ^3^ Department of Thoracic Surgery Cancer Hospital of Dalian University of Technology, Liaoning Cancer Hospital and Institute Shenyang China

**Keywords:** frizzled, glycosylation, post‐translational modification, protein maturation, Wnt signalling pathway

## Abstract

Wnt signalling is a highly conserved signalling pathway that plays an important role in a variety of biological processes. Frizzled (Fzd) family proteins are receptors for Wnt ligands. The physiological processes involved in mature trafficking of Fzd proteins remain elusive. Here, we identified asparagine residues 59 and 144 as the N‐glycosylation modification sites of Fzd4. Sequence analysis of Fzd4 in different species showed that the two asparagine residues were highly conserved. N‐glycosylation modification of Fzd4 is indispensable for its maturation and transport to the plasma membrane. N‐glycosylation modification enhances the stability of Fzd4 and is also necessary for Fzd4 activity, which promotes Fzd4 interaction with Wnt ligands and co‐receptor Norrin. Knockout of Fzd4 in the non‐small cell lung cancer (NSCLC) cell line A549 followed by replenishment of Fzd4 glycosylation site mutants inhibited the growth and migration ability of A549 cells in vitro and in vivo. In summary, we identified N‐glycosylation modification sites of Fzd4. N‐glycosylation modification of Fzd4 is necessary for its stability and activity. When N‐glycosylation modification is absent, Fzd4 cannot mediate the Wnt/β‐catenin signalling pathway, which can inhibit the proliferation and migration of NSCLC and provide new targets and strategies for the treatment of NSCLC.

## 1 Introduction

Generally, proteins need to be processed in the Golgi after ribosome synthesis and released to become biologically active mature proteins [1, 2, 3]. This post‐translational modification (PTM) process is not a simple process, and it usually needs to be regulated by a variety of enzymes and other factors. It has been found that there are many kinds of PTMs of proteins in eukaryotic cells, of which the relatively common types are: methylation, acylation, ubiquitination, glycosylation, and phosphorylation [4, 5, 6, 7, 8]. Among these modifications, glycosylation modification plays an important role in protein function and structure formation. Glycosylation refers to the process of transferring sugar chains to peptide chains under the action of enzymes after protein precursor synthesis, which in turn can form glycosidic bonds between amino acid residues at specific positions on the peptide chain [9]. It has been found that more than 50% of human proteins are glycosylated, and this modification plays a very important role in both cellular (cell–cell interactions, signalling, immune responses) and protein levels (molecular recognition, protein folding) [10, 11, 12].

Wnt signalling pathway is a ubiquitous signalling pathway in multicellular organisms, which plays an important role in various biological processes such as cell proliferation, development, metabolism, and stemness. Wnt signalling pathways are divided into two types: canonical Wnt pathway (β‐catenin dependent pathway) and non‐canonical Wnt pathway (β‐catenin independent signalling pathway) [13]. The canonical Wnt/β‐catenin pathway has become one of the current research hotspots in developmental biology and tumour molecular biology [14]. Fzd protein is a very important class of receptors containing seven transmembrane domains in the Wnt signalling pathway and belongs to the G protein‐coupled receptor family (GPCRs) F class, which are both secreted glycoproteins [15]. Fzd contains a signal peptide and a cysteine‐rich CRD domain extracellularly. In addition, structurally, the Fzd receptor contains three extracellular and three intracellular loops and an intracellular C‐terminus. Fzd interacts with Wnt through its extracellular CRD domain, and further regulation occurs through intracellular binding to cytoplasmic partners, allowing the Wnt‐Fzd receptor complex to accumulate and promote its endocytosis and downstream signalling [16]. At present, the biological function of Fzd has been elucidated in various cancers and normal development, including cancer cell proliferation, migration, and invasion, angiogenesis, stemness and chemoresistance after cancer recurrence [17]. However, the physiological processes involved in Fzd maturation remain to be investigated. In this study, we identified the N‐glycosylation site of Fzd4, investigated the effect of N‐glycosylation on Fzd4 function, further supplemented the maturation process of Fzd protein, and also provided new targets and strategies for the treatment of NSCLC.

## 2 Methods and Materials

### 2.1 Animal Feeding and Tumour Formation in Nude Mice

Animals were purchased from Beijing HFK Bioscience CO., LTD. A549 cells were trypsinized, fully digested into individual cells, and washed with PBS before being resuspended in PBS. Designed cell numbers and viability were determined using trypan blue. A549 cells were injected subcutaneously into nude mice (Beijing HFK Bioscience CO., LTD) at a density of 1 × 10^6^ cells per site. Tumour volumes were measured at regular intervals beginning 1 week after tumours formed subcutaneously in nude mice. Tumour volume was calculated using the formula: 1/2 (Length × Width^2^). Mice maintenance and treatments described were approved by the Ethics Committee of Xiyuan Hospital, China Academy of Chinese Medical Sciences (approval no.: 2019XLC018‐3).

### 2.2 Cell Lines and Cell Culture

HEK293T, A549 cell lines were purchased from ATCC (Shanghai, China). Both cell lines were cultured in DMEM‐high glucose supplemented with 10% FBS (fetal bovine serum) and 100 mg/mL penicillin/streptomycin/glutamine (Gibco). All cell lines were incubated at 37°C in a humidified incubator with 5% CO_2_.

### 2.3 Clones and Constructs

The plasmids expressing Flag‐tagged full‐length human Fzd4, all Flag‐tagged Fzd4 mutants, were constructed in the customised Lenti‐EF1α‐puro vector. The plasmid containing sgFzd4 was constructed in the Lenti‐Cas9‐puro vector. All plasmids were transformed in *E. coli* NEB 5‐alpha for amplification and extracted by the OMEGA Endo‐free Plasmid Mini Kit. The concentrations of all plasmids were determined by the Thermo Nanodrop 2000.

### 2.4 Cell Transfection

Cells were transfected using Invitrogen Lipofectamine 2000. Transfection was performed according to the product instructions. Briefly, plasmids were diluted using DMEM and subsequently mixed with Lipofectamine 2000. Complexes were incubated at room temperature (RT) for 20 min, added to HEK293T cells, and 48 h later, cells were collected for subsequent experiments.

For lentivirus production, psPAX2 vector (for packaging, Addgene) and pCMV‐VSV‐G (for enveloping, Addgene) and desired customised Lenti‐EF1α‐puro plasmids, Lenti‐Cas9‐puro sgFzd4 or other lentiviral vector‐based plasmids were co‐transfected in HEK293T cells by the ratio of 5:1:5 in mass (ng). After transfection for 24 h, the medium was replaced with fresh medium to produce virus‐containing conditioned medium. The virus‐containing conditioned medium was collected after 72 h and followed by concentration if necessary. For lentiviral infection, the cell culture was added 0.5–1 mL virus‐containing conditioned medium or 0.1–0.5 mL virus concentrate for 48 h. After 48 h, the culture was refreshed and a certain selection drug was added for 0resistance selection.

### 2.5 Westernblot

Cells were lysed by lysis buffer (25 mM Tris–HCl, 150 mM NaCl, 0.5% CA630, pH = 7.4) containing protease inhibitor cocktail (Roche) and the amount of total protein was subsequently determined by BCA assay and SDS‐PAGE was performed using polyacrylamide gels. Afterwards, PVDF membranes were used for protein transfer. Membrane blocking and antibody incubation were performed using 5% skimmed milk. For western blot, all primary antibodies were used at 1:1000 dilution and all secondary antibodies were used at 1:5000 dilution. Primary antibody information was as follows: Flag (14,793, Cell Signalling technology), GAPDH (92,301, Cell Signalling technology), β‐catenin (8480, Cell Signalling technology), Wnt3a (C64F2, Cell Signalling technology), Norrin (A1964, ABclonal). The plasma membrane separation method: Cells were first lysed using (25 mM Tris–HCl, 150 mM NaCl, 0.015% digitonin, pH = 7.4) containing protease inhibitor cocktail (Roche) lysis buffer for 10 min. Centrifugation was performed at 300 × g for 5 min after completion of lysis, and the pellet in the solution after completion of centrifugation was the cell membrane fraction.

### 2.6 SNAP Protein Staining

For SNAP protein staining, plasmids were transfected into HEK293T cells for 48 h before subsequent experiments. Cells were then incubated with SNAP‐Surface 549 (NEB) dye or SNAP‐Cell Oregon Green (NEB) for 30 min at 37°C. Subsequently, cells were washed three times for 5 min each with cold PBS. They were fixed in 4% paraformaldehyde (Aladdin) after washing with PBS. Hoechst was used to label nuclei [18].

### 2.7 Immunofluorescence

HEK293T cells were grown on poly‐L‐lysine‐coated glass coverslips. Cells were transfected for 24 h and fixed in 4% paraformaldehyde (Sigma) for 30 min at RT. The fixed cells were washed twice with cold PBS, permeabilized and blocked with 0.1% Triton X‐100/5% BSA/PBS for 30 min. The permeabilized cells were incubated overnight at 4°C in the dark with primary antibody (1:200 dilution) and then washed with PBS 5 times, followed by incubation with secondary antibody at RT in the dark for 1 h (1:1000) and 5 times washed with PBS. Hoechst (Thermo) was next used to stain nuclei for 10 min, followed by 3 times washed with PBS. Samples were observed using inverted confocal microscopy (DM6000CS, Leica).

### 2.8 Dual‐Luciferase Reporter System

For the dual‐luciferase reporter system, experiments were performed using HEK293T cell lines stably expressing firefly luciferase and Renilla luciferase. HEK293T cells were plated in 24‐well plates and subsequently transfected the following day using Lipofectamine 2000. Experiments were performed using a dual‐luciferase reporter assay kit (Sigma). Each experiment was repeated in triplicate. Chemical reflectance intensity was measured by a microplate reader.

### 2.9 MTT Assay for Cell Proliferation

Cells were plated onto 96‐well plates at a density of 500 cells per well and cultured for 1–8 days. Thiazolyl blue tetrazolium (Aladdin) was added to each well at regular intervals to a final concentration of 0.5 mg/mL, and plates were incubated for an additional 4 h at 37°C. Following incubation, all medium was removed, and 100 μL DMSO was added to each well. Plates prepared for testing were then analysed by a microplate reader at OD490. Growth curves were plotted by days based on OD490 values.

### 2.10 Surface Biotin Labeling Assay

Cell surface proteins were biotinylated using Sulfo‐NHS‐SS‐Biotin (APExBIO) according to the manufacturer's protocol. Specifically, cells were incubated with Sulfo‐NHS‐SS‐Biotin medium containing 1 mg/mL for 30 min after the completion of cell transfection. Following incubation, cells were lysed and lysates collected. Then biotinylated proteins were enriched by Pierce Streptavidin Agarose. Enrichment was performed overnight at 4°C. Western blot was performed the following day.

### 2.11 Colony Formation Assay

Cells were plated on six‐well plates at a density of 1000 cells per well and cultured for 14 days. Fresh medium was changed regularly. Cells were subsequently fixed using 4% paraformaldehyde for 30 min. Cells were washed three times for 5 min each with PBS after fixation. Staining was then performed using 0.1% crystal violet solution for 5 min, and cells were washed 3 times with PBS for 5 min each. Photographic recordings were taken using a digital camera.

### 2.12 Realtime‐PCR

Total cellular RNA was purified by RNA isolation kit (Sango Biotech). CDNA was generated using MonScript RTIII and dsDNase (Monad) according to the kit instructions. Quantitative RT‐PCR was performed using MonAmp ChemoHS qPCR Mix (Monad). The reaction mixture was incubated at 50°C for 15 min and then at 95°C for 5 min, followed by 32 cycles of PCR with the following temperature profile: 95°C for 15 s, 60°C for 30 s, 72°C for 1 min. Gene expression values were normalised to those of GAPDH. Primer details are as follows: AXIN2: F: AGCCAAAGCGATCTACAAAAGG. R: AAGTCAAAAACATCTGGTAGGCA. cMYC: F: GTCAAGAGGCGAACACACAAC. R: TTGGACGGACAGGATGTATGC. CD44: F: CTGCCGCTTTGCAGGTGTA. R: CATTGTGGGCAAGGTGCTATT. GAPDH: F: GGAGCGAGATCCCTCCAAAAT. R: GGCTGTTGTCATACTTCTCATGG.

### 2.13 Transwell Assay

1 × 10^5^ cells were inoculated into the upper chamber of the transwell and, after 72 h of incubation, the transwell was removed, the culture medium in the chamber was discarded, and the upper non‐migrated cells were gently removed using a cotton swab. Cells were subsequently fixed using paraformaldehyde for 30 min. Staining was performed using crystal violet dye for 15 min, washed with PBS, and observed using a microscope.

### 2.14 Data Statistics and Analysis

Statistical analysis was performed using GraphPad Prism 8.0 software. A two‐tailed student's *t*‐test was used to compare the two sets of data. For one factor, comparisons were made between three or more groups using one‐way ANOVA. For two factors, comparisons were made between three or more groups using two‐way ANOVA. Experimental results are presented as mean ± SD. A *p*‐value < 0.05 was considered statistically significant.

## 3 Results

### 3.1 Identification of Fzd4 Glycosylation Modification Sites

**FIGURE 1:**
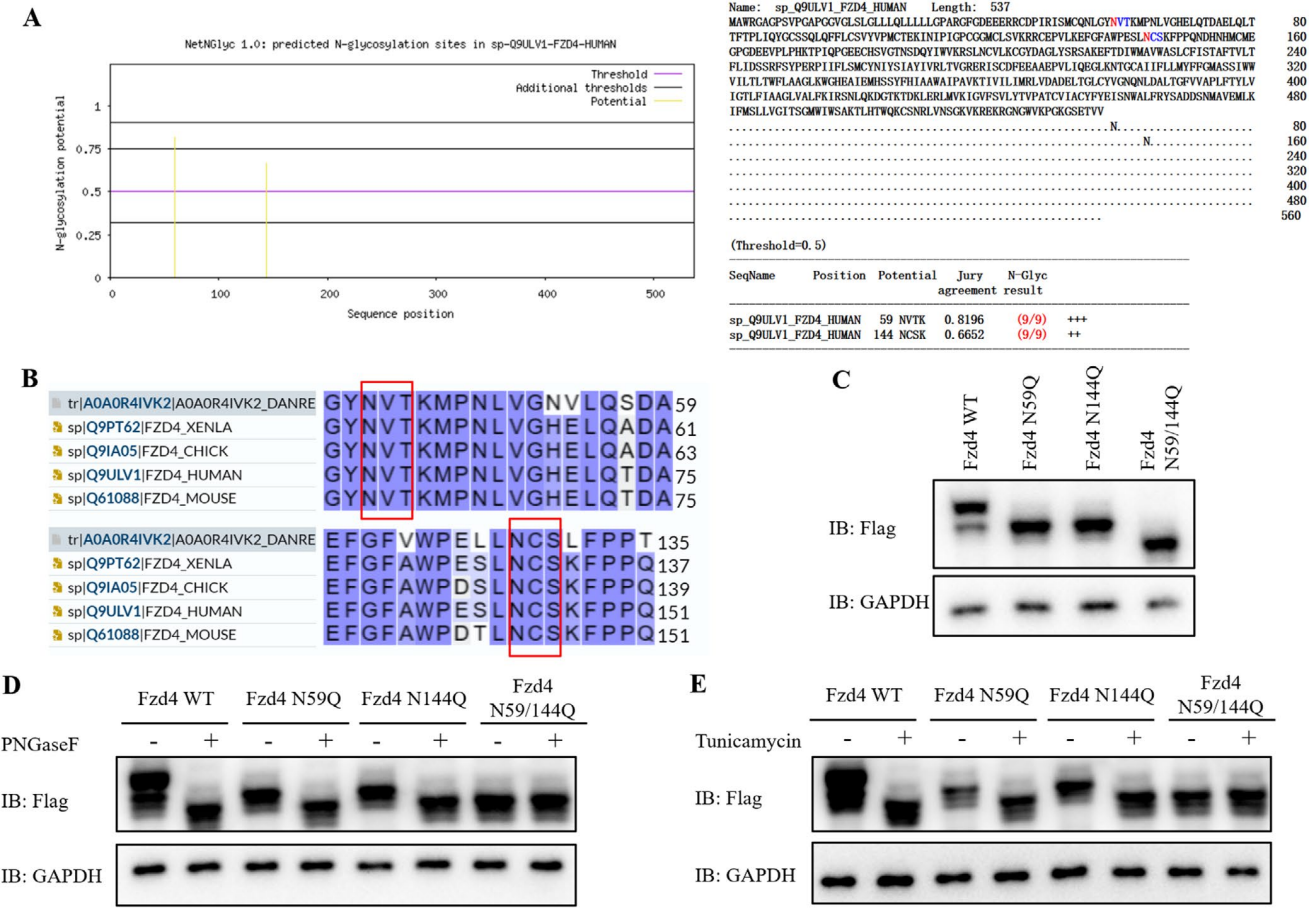
Identification of N‐glycosylation modification sites of Fzd4 protein. (A) NetNGlyc 1.0 server N‐glycosylation prediction website prediction of N‐glycosylation modification sites of Fzd4. (B) Comparative results of Fzd4 sequence conservation between species. (C) Western blot results for Fzd4 WT, Fzd4 N59Q, Fzd4 N144Q, and Fzd4 N59/144Q expression. (D) Western blot results of Fzd4 WT, Fzd4 N59Q, Fzd4 N144Q and Fzd4 N59/144Q after PNGase F treatment. (E) Western blot results of Fzd4 WT, Fzd4 N59Q, Fzd4 N144Q and Fzd4 N59/144Q treated with Tunicamycin.

The N‐glycosylation sites of Fzd4 were predicted using the NetNGlyc 1.0 Server N‐glycosylation prediction website, respectively, and the glycosylation predicted by Fzd4 was N59 and N144 sites (Figure 1A). Subsequent sequence comparisons revealed that both predicted glycosylation sites were highly conserved between species (Figure 1B). Subsequently, Flag‐tagged Fzd4 plasmids were constructed, and Fzd4 N59Q (mutated N59 to Q59), Fzd4 N144Q (mutated N144 to Q144), and Fzd4 N59/144Q (N59, N144 double mutated plasmids) plasmids with mutations at N59 and N144 sites. The constructed plasmids were transfected into HEK293T cells by transient transfection. Figure 1C showed that the gel protein displacement of Fzd4 mutant protein at N59 and N144 glycosylation sites changed as compared with Fzd4 WT, and the gel protein displacement changed as compared with Fzd4 WT. When the Fzd4 N59Q and N144Q proteins were treated with deglycosidase (PNGaseF), their protein gel shifts were consistent with the changes in the gel shifts of the Fzd4 WT protein treated with glycosidase, both of which subsided, and only the Fzd4 N59/144Q protein was treated with PNGaseF compared with the protein gel shifts without glycosidase (Figure 1D). The same results were obtained with Tunicamycin treatment, an N‐glycosylation modification inhibitor (Figure 1E). Thus, we identified that both N59 and N144 were N‐glycosylation sites of Fzd4.

### 3.2 Effect of N‐Glycosylation Modification on Fzd4 Protein Transport

**FIGURE 2:**
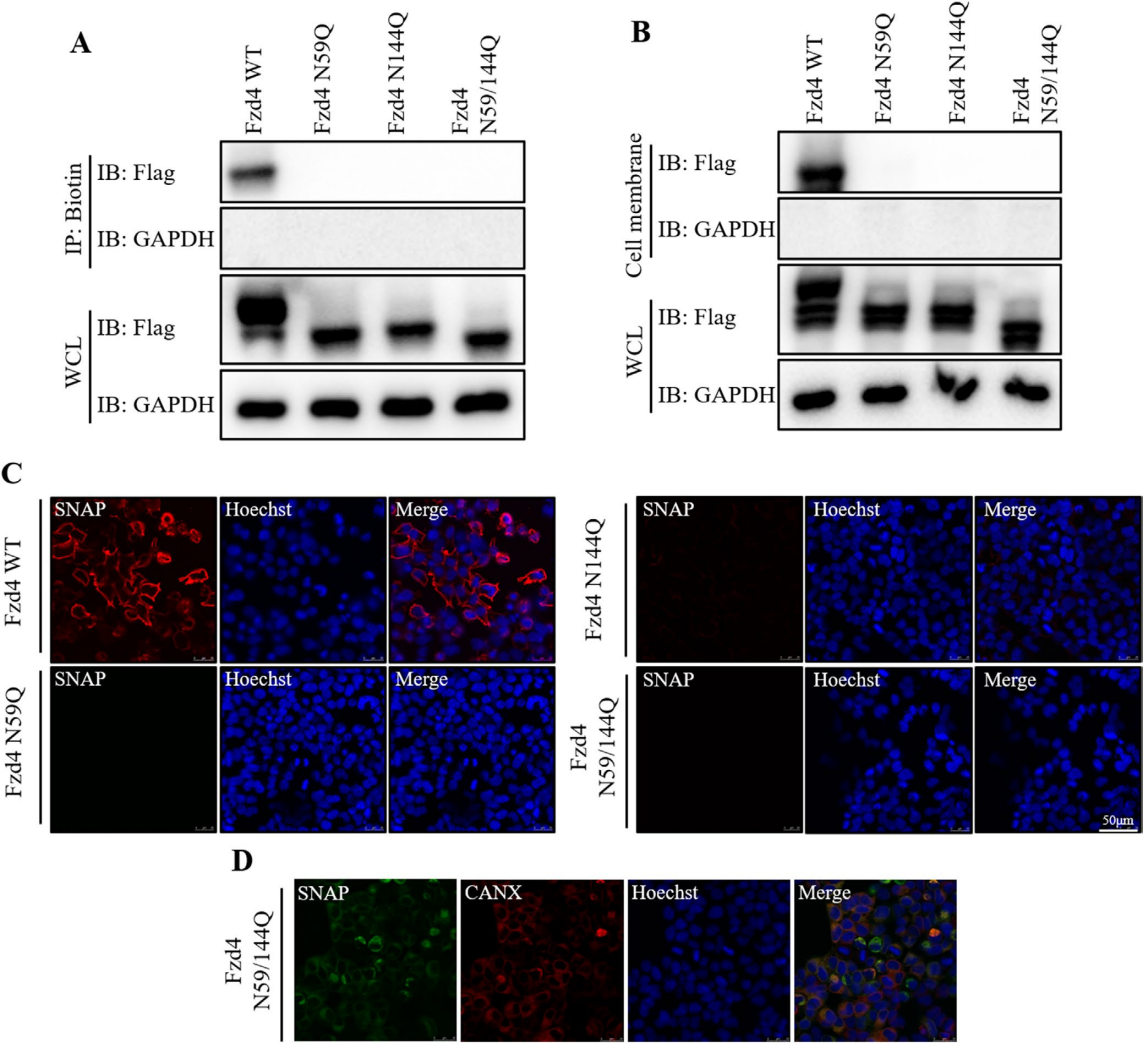
N‐glycosylation modification impacts Fzd4 maturation trafficking to cell membranes. (A) Biotin‐labelled cell membrane protein pull‐down assay results. (B) Western blot assay results for plasma membrane separation. (C) Representative pictures of SNAP‐labelled staining. (D) Co‐localisation experiments of Fzd4 N59/144Q with markers of the ER (CANX). Scale bar: 50 μm.

To further investigate the effect of N‐glycosylation modifications on Fzd4 function, we first expressed Fzd4 and Fzd4 glycosylation mutants in HEK293T cells. Biotin‐labelled membrane proteins were labelled using biotin, and subsequent pull‐down experiments were performed on Biotin‐labelled membrane proteins using Streptavidin. From the experimental results, we found that only Fzd4 WT was able to detect the presence at the cell membrane, while none of the glycosylation mutations detected the presence at the cell membrane (Figure 2A). We subsequently got the same results using plasma membrane separation experiments (Figure 2B). To further validate our conclusions, we constructed a SNAP‐tagged Fzd4 plasmids, transfected the SNAP‐tagged plasmids into HEK293 cells, and stained them with dyes that did not enter the cell membrane, and found that only Fzd4 WT could be present on the cell membrane (Figure 2C). Because the Fzd4 mutant was not able to mature to transport to the cell membrane, we speculated that it might be misfolded in the endoplasmic reticulum (ER). We subsequently further confirmed this conjecture by co‐localization experiments with markers of the endoplasmic reticulum (CANX) (Figure 2D). We therefore concluded that glycosylation modification of Fzd4 was capable of affecting mature trafficking of Fzd4 to the cell membrane.

### 3.3 Effect of N‐Glycosylation Modification on Fzd4 Protein Stability

**FIGURE 3:**
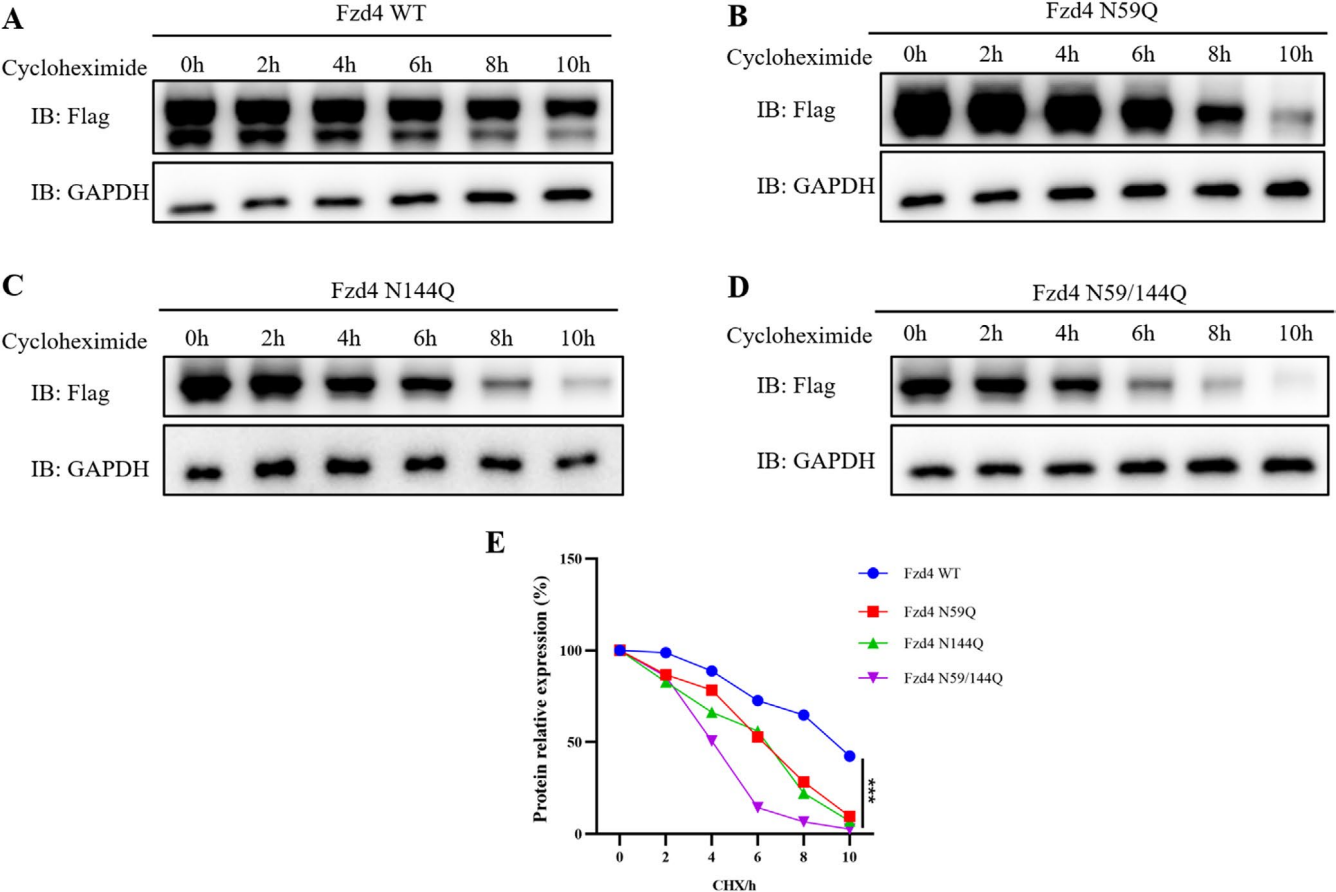
Stability experiments for Fzd4 and glycosylation mutants. (A–D) Cycloheximide (ribosomal inhibitor) was used to inhibit protein synthesis of Fzd4 and glycosylation mutants, and their protein stability was tested by Western blot. (E) Data of (A–D) were quantified and normalised with 0 h. *** *p* < 0.001.

Fzd4 WT and N‐glycosylation site mutant plasmids were transfected by transient transfection in HEK293T cells for overexpression. Cycloheximide (ribosomal inhibitor) was added for protein synthesis inhibition, and then protein collection was performed according to the time gradient. Glycoprotein degradation was observed and analysed by Western blot (Figure 3A–D). We can find that wild‐type Fzd4 has a very slow turnover rate of protein expression after the addition of ribosomal inhibitors, which is relatively stable (Figure 3A). Whereas Fzd4 mutated at glycosylation sites N59 and N144 was less stable and degraded at a faster rate (Figure 3B,C). Fzd4 showed the fastest degradation rate after mutation at both N59 and N144 sites (Figure 3D,E). The above results indicated that the presence of N‐glycosylation modifications increased the stability of Fzd4.

### 3.4 Effect of N‐Glycosylation Modification on Fzd4 Protein Function

**FIGURE 4:**
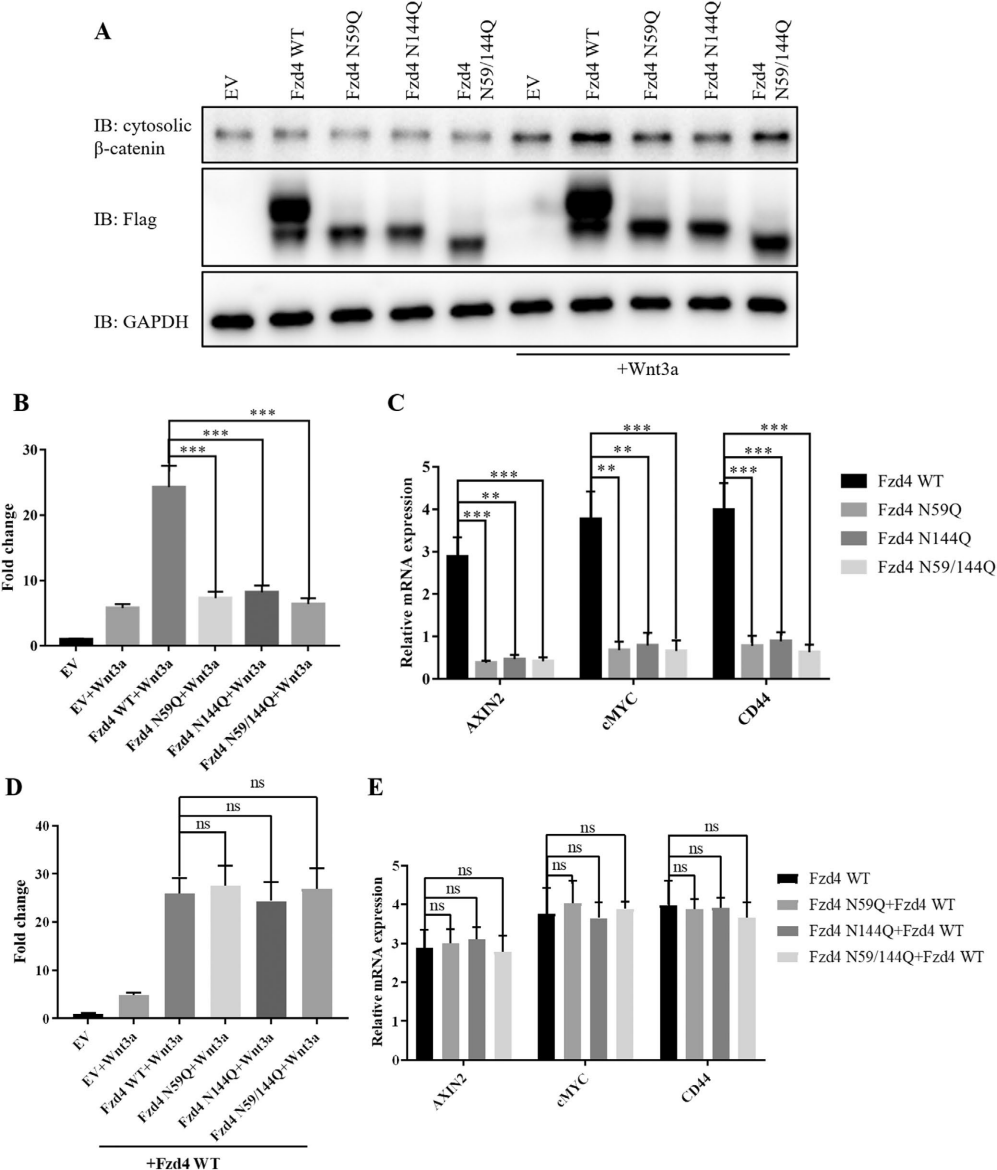
Effect of N‐glycosylation modification on canonical Wnt signalling mediated by Fzd4. (A) Western blot was used to detect changes in intracellular β‐catenin content. (B) The dual‐luciferase reporter system was used to examine the effect of N‐glycosylation modification on the canonical Wnt signalling pathway mediated by Fzd4. (Data represent triplicate experiments, ** *p* < 0.01, *** *p* < 0.001). (C) RT‐PCR detection of Wnt target genes was conducted to investigate the effect of N‐glycosylation modification on the Fzd4‐mediated canonical Wnt signalling pathway. (Data represent triplicate experiments, ** *p* < 0.01, *** *p* < 0.001). (D) The dual‐luciferase reporter system was used to examine the dominant negative effect of the Fzd4 N‐glycosylation loss mutant. (Data represent triplicate experiments, ns: not significant). (E) RT‐PCR detection of Wnt target genes was conducted to investigate the dominant negative effect of the Fzd4 N‐glycosylation loss mutant. (Data represent triplicate experiments, ns: not significant).

To further investigate the effect of N‐glycosylation modification on Fzd4 function, we examined intracellular β‐catenin content using Western blot. As a result, we found that Fzd4 WT was able to mediate the Wnt‐β‐catenin signalling pathway normally, thereby allowing accumulation of intracellular β‐catenin. On the other hand, glycosylation mutants failed to increase intracellular β‐catenin content (Figure 4A). We subsequently validated this using a dual luciferase reporter system and obtained similar results, with Fzd4 WT significantly activating the Wnt‐β‐catenin signalling pathway, whereas glycosylation mutants did not (Figure 4B). Similar results were obtained by detecting Wnt‐β‐catenin signalling pathway target genes using RT‐PCR (Figure 4C). Subsequently, we co‐transfected Fzd4 WT with Fzd4 mutants to investigate whether there was a dominant negative effect of Fzd4 mutants. The results showed that the Fzd4 mutant did not affect the exertion of Fzd4 WT function (Figure 4D,E). Based on these results, both N59 and N144 are potent glycosylation sites for Fzd4 and also affect canonical Wnt signalling.

### 3.5 Effect of N‐Glycosylation Modification on Fzd4 Protein Structure

**FIGURE 5:**
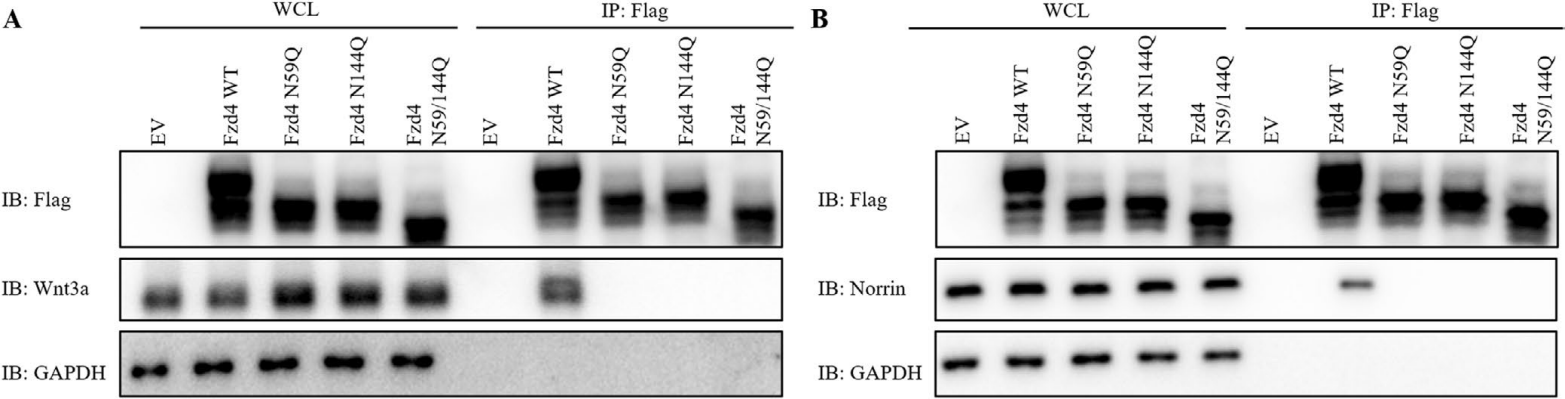
Effect of Glycosylation Modification on Fzd4 Protein Structure. (A, B) Interaction detection of Fzd4 and its glycosylation mutants with Wnt3a ligands and the co‐receptor Norrin.

Fzd4 protein can turn on transduction of downstream signalling pathways through interaction of the co‐receptor Norrin with the Wnt ligand. We subsequently investigated the interaction of Fzd4 wild type and glycosylation mutants with the co‐receptor Norrin and the Wnt3a ligand. By Co‐ip assay we found that Fzd4 wild type showed significant interaction with Wnt3a and Norrin, while glycosylation mutants showed significantly attenuated interaction with Wnt3a and Norrin (Figure 5A,B). We therefore hypothesised that N‐glycosylation modification can affect the protein impact of Fzd4 by affecting the maturation of Fzd4 and thus affect the exertion of the normal physiological function of Fzd4.

### 3.6 The Test of N‐Glycosylation Modification on Fzd4 Function In Vitro

**FIGURE 6:**
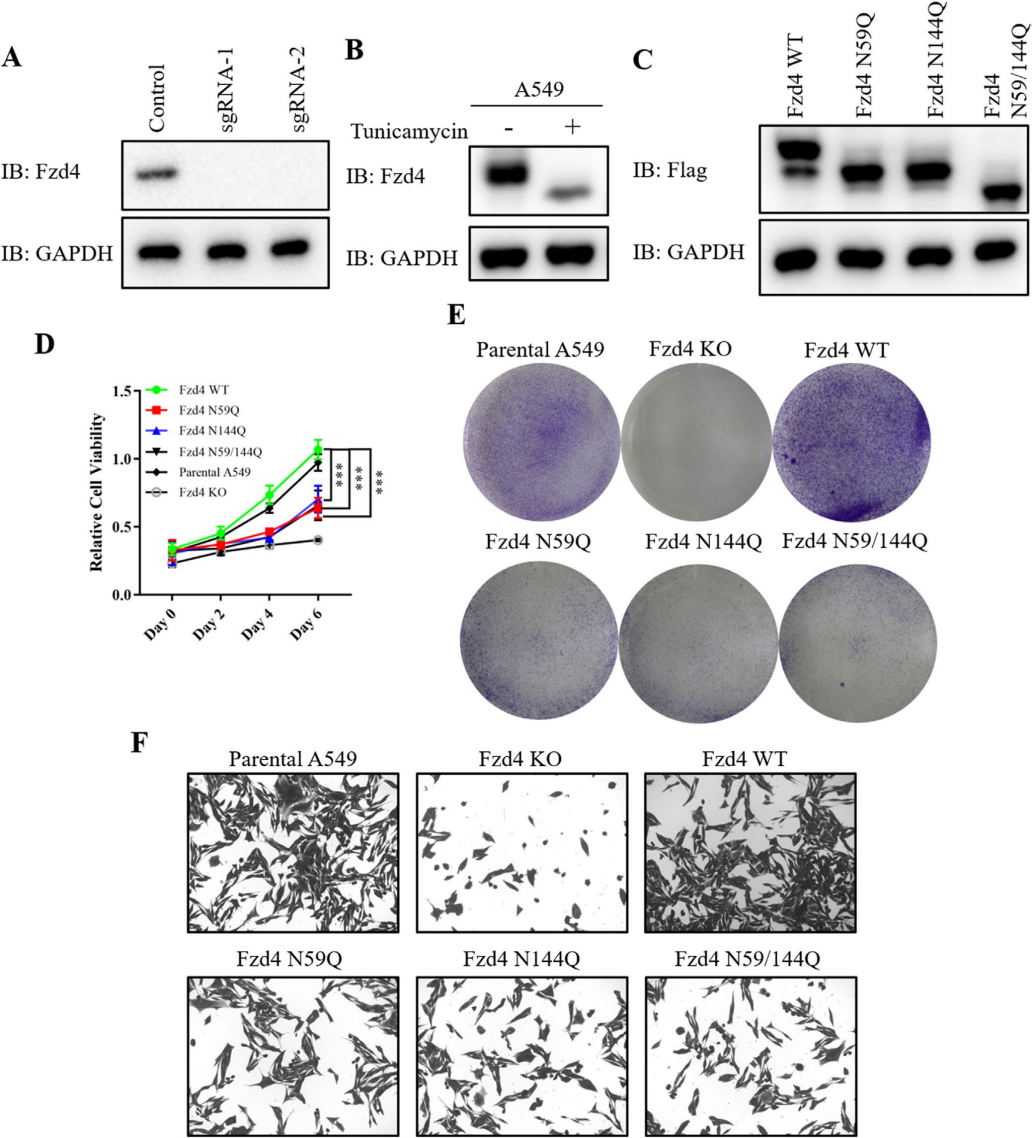
Validation of N‐glycosylation modifications on Fzd4 function in vitro. (A) Validation of Western blot results for knockout of Fzd4 in A549 cells. (B) Western blot results for expression of Fzd4 in A549 cells with tunicamycin treatment. (C) Western blot results of Fzd4 and its glycosylation mutants replenished after knockout of Fzd4 in A549 cells. (D) MTT assay of A549 cell line supplemented with Fzd4 and its glycosylation mutants was used to detect the proliferation ability of the cells. (Data represent triplicate experiments, *** *p* < 0.001). (E) Cloning assays of A549 cell lines supplemented with Fzd4 and its glycosylation mutants were used to detect the proliferation ability of the cells. (F) Transwell assay of A549 cell line supplemented with Fzd4 and its glycosylation mutants was used to detect the migration and invasion of the cells.

It has been reported that Fzd4 played a key role in NSCLC. Subsequently, we performed experiments using A549 cells. Firstly, we knocked out Fzd4 in A549 cells (Figure 6A). A549 cells were treated with tunicamycin, and the results showed that the endogenous Fzd4 band also showed a decrease, indicating that endogenous Fzd4 also underwent N‐glycosylation modification (Figure 6B). We subsequently replenished Fzd4 wild type and glycosylation mutants (Figure 6C). By MTT assay, we found that the growth of A549 cells was significantly inhibited after the knockout of A549 cells, and this growth inhibition was rescued after the replenishment of Fzd4 wild type, but the replenishment of glycosylation mutants did not rescue this phenomenon (Figure 6D). Cloning experiments similarly confirmed this conclusion (Figure 6E). At the same time, the replete glycosylation mutant also significantly inhibited the migration and invasion ability of A549 cells (Figure 6F). The above results showed that N‐glycosyl modification was essential for the function of Fzd4 in NSCLC and could affect the growth, migration, and invasion of NSCLC.

### 3.7 Validation of Glycosylation Modification on Fzd4 Function In Vivo

**FIGURE 7:**
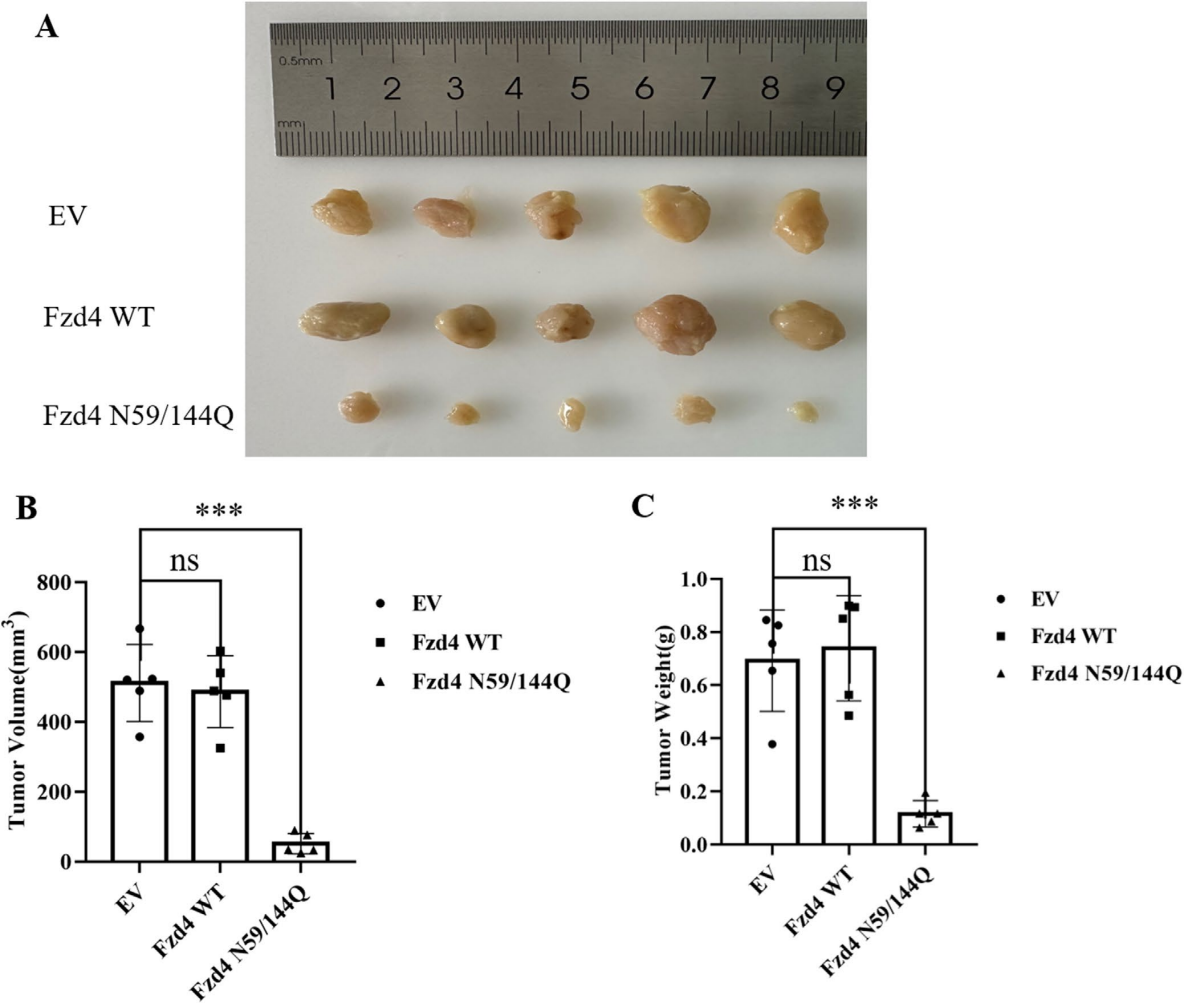
Validation of N‐glycosylation modification on Fzd4 function in vivo. (A) Tumour images formed using A549 cells. (The number of nude mice in each group of experiments was 5). (B) Volume of tumour formed in (A) (Data represent five replicate experiments, ** *p* < 0.01, *** *p* < 0.001). (C) Tumour weight formed in A. (Data represent five replicate experiments, *** *p* < 0.001).

We subsequently further validated the effect of N‐glycosyl modification on Fzd4 function using xenografts in nude mice. We xenografted different A549 cell lines into nude mice and found that A549 cells replenished with Fzd4 WT rapidly formed tumours, whereas A549 cells replenished with glycosylated mutants formed tumours at a slower rate or even failed to form tumours (Figure 7A). At the same time, tumours formed by A549 cells replenished with Fzd4 WT grew significantly faster than tumours formed by A549 cells replenished with glycosylated mutants (Figure 7B). Finally, A549 cells supplemented with Fzd4 WT formed significantly heavier tumours than A549 cells supplemented with glycosylation mutants (Figure 7C). The above results suggested that N‐glycosylation modification can also have an important impact on the exertion of Fzd4 function in vivo.

## 4 Discussion

Oligosaccharide structures on the cell surface have many biological functions, and they interacted with the extracellular environment through adhesion to cells, interaction between macromolecules and pathogen invasion [19]. At the same time, glycans associated with cell surface receptors and proteins can not only directly regulate protein function and the conduction of related signals, but also change the endocytosis of glycoproteins and the half‐life of cell surfaces by binding to multivalent lectins [20]. Glycan structures on newly synthesised glycoproteins were also essential for protein secretion and maturation, and they affected the correct folding of proteins and provided ligands for lectin partners, thereby contributing to endoplasmic reticulum (ER) quality control monitoring and mediating transport throughout the secretory pathway and targeted trafficking of selective proteins [21, 22]. In addition, glycan structures can also regulate cytoplasmic and nuclear functions and play an important role in immune surveillance, inflammatory response, autoimmunity, hormone action and tumour metastasis [23].

In the ER, when chaperones cadherin (CNX) and calreticulin (CRT) assist newly synthesised glycoproteins to fold in the ER, they firstly bind oligosaccharides with 14 residues to proteins to form glycoprotein nascent chains under the action of oligosaccharyl transferase (OST), and then glycoproteins in the monoglycosylated form bind to the CNX and CRT domains to promote their folding [24]. If a protein is able to fold correctly to its native conformation, it would be released from the ER via the protein secretory pathway. However, if the protein folding is incomplete, the exposed hydrophobic region is recognised by glycoprotein glycosyltransferases (UGGT), which readd the removed glucose to the glycoprotein of the UDP‐glucose donor, which generates a monoglycosylated N‐glycan on the glycoprotein and again promotes binding to CNX/CRT, which in turn refolds. Therefore, proteins may undergo multiple CNX/CRT cycles before reaching their native conformation for correct folding of the protein [25]. However, if the protein remains unable to fold correctly or appears misfolded, the unfolded protein would enter the targeted ER‐associated degradation (ERAD) pathway. ERAD‐targeted proteins undergo sequential de‐mannosylation with the aid of the ER‐resident mannosidase EDEM. Then, the demannosylated ERAD substrate is recognised and bound by XTP3‐B and OS‐9, thus preventing their aggregation, and then further targeted for ubiquitin recognition labeling and degradation by the proteasome [26, 27]. In addition, when incorrectly folded proteins accumulate continuously, the unfolded protein response (UPR) is triggered, and the UPR processes misfolded proteins by coordinately reducing protein expression, increasing chaperone expression, and increasing protein degradation associated with the ER to eliminate misfolded proteins, thereby restoring adaptive responses to ER homeostasis [28, 29]. In this study, we showed that Fzd4 was not able to undergo glycosylation modification and was not able to successfully mature and transport to the cell membrane, and in combination with previous studies we suspected that Fzd4 that was not able to mature will accumulate in the endoplasmic reticulum and thus be degraded through ERAD. Kaykas et al. [30] reported that Fzd mutations that accumulate in the ER form oligomers with Fzd WT, which leads to the same aggregation of Fzd WT that is not able to function in the ER and shows a strong dominant negative effect. In this study, we tested whether the Fzd4 mutant, which is unable to undergo N‐glycosylation, has a dominant negative effect, and showed that the Fzd4 mutant does not affect the function of the Fzd4 WT, which may be caused by the importance of N‐glycosylation for the Fzd4 conformation, rendering the Fzd4 N‐glycosylation loss mutant unable to bind to the Fzd4 WT.

Fzd protein acts as an essential transmembrane receptor in the Wnt signalling pathway, and this receptor is importantly regulated by glycosylation modification, which is an important process in protein post‐translational modification, and this modification plays a very important role in protein maturation, secretion, and transport [31]. Sugar chain modification has been shown to promote the maturation and transport of Fzd family proteins on membranes. The study focused on smoothened (Smo), the only protein of the Frizzled protein family, a non‐Fzd protein, and found that only one of the seven predicted N‐glycan sites of Drosophila Smo was essential and showed that the loss of N‐glycosylation at this site disrupts Smo transport and weakens its signalling capacity [32]. As an important membrane receptor of the Wnt signalling pathway, the successful trafficking of Fzd is important for the signalling pathway, so we wanted to investigate whether glycosylation also affects Fzd maturation and trafficking. Therefore, in this study, we predicted the glycosylation site of Fzd4 and mutated the predicted asparagine residues 59 and 144, which were experimentally found to be able to undergo glycosylation modification, and both glycosylation modifications could affect the mature transport of Fzd4 and the exertion of the physiological function of Fzd4. When Fzd4 is not able to undergo glycosylation modification, Fzd4 is able to mature and translocate to the cell membrane for function. Meanwhile, the transduction of the Wnt‐Fzd4‐β‐catenin signalling pathway was inhibited when Fzd4 was not able to undergo glycosylation modification, as confirmed by in vitro and in vivo assays.

Fzd5 has been reported to play a critical role in a variety of tumours. Zheng et al. reported that Fzd5 is mandatory for the survival of RNF43‐mutated PDAC [18]. Fzd7 also plays a key role in the development of breast cancer [33]. NSCLC is one of the most aggressive cancers and is characterised by a very poor prognosis [34, 35]. Current treatments for NSCLC are limited. Several literature reports have found that Fzd4 plays a key role in the development of NSCLC [36, 37]. In this study, we found that the knockout of Fzd4 in the NSCLC cell line A549 did result in inhibition of A549 cell proliferation and invasion. The effects of Fzd4 knockout could be compensated by replenishing Fzd4 wild‐type, but A549 cells could not grow and proliferate normally in vitro and in vivo in replenishing Fzd4 glycosylation mutants. Therefore, this study also provided a new therapeutic target and idea for the treatment of NSCLC.

## 5 Conclusion

The N‐glycosylation modification site of Fzd4 was identified and the N‐glycosylation modification function of Fzd4 was explored in this study. N‐glycosylation modification is essential for the mature transport of Fzd4 to the cell membrane and can affect the exertion of Fzd4 function. At the same time, validating the effect of N‐glycosylation modification on Fzd4 function in vitro and in vivo can provide new therapeutic targets and ideas for the treatment of NSCLC.

## Author Contributions

**Tianyi Ji:** conceptualization (equal), data curation (equal), writing – original draft (lead), writing – review and editing (lead). **Xiangying Li:** methodology (equal), software (lead), writing – original draft (supporting), writing – review and editing (supporting). **Jiachen Li:** data curation (equal), formal analysis (equal), methodology (lead), writing – original draft (supporting). **Guan Wang:** conceptualization (lead), funding acquisition (lead), investigation (lead), supervision (lead), writing – review and editing (equal).

## Conflicts of Interest

The authors declare no conflicts of interest.

## Data Availability

The data that support the findings of this study are available from the corresponding author upon reasonable request.
